# Is endocan a novel potential biomarker of liver steatosis and fibrosis?

**DOI:** 10.2478/jomb-2019-0042

**Published:** 2020-09-02

**Authors:** Aleksandra Klisić, Nebojša Kavarić, Ludovico Abenavoli, Verica Stanišić, Vesna Spasojević-Kalimanovska, Jelena Kotur-Stevuljević, Ana Ninić

**Affiliations:** 1 University of Montenegro, Faculty of Medicine, Primary Health Care Center, Podgorica, Montenegro; 2 University Magna Graecia, Department of Health Sciences, Catanzaro, Italy; 3 Clinical Center of Montenegro, Podgorica, Montenegro; 4 University of Belgrade, Faculty of Pharmacy, Department for Medical Biochemistry, Belgrade

**Keywords:** endocan, inflammation, liver steatosis, liver fibrosis, cardiovascular disease, endokan, inflamacija, steatoza jetre, fibroza jetre, kardiovaskularne bolesti

## Abstract

**Background:**

Studies that evaluated endocan levels in nonalcoholic fatty liver disease (NAFLD) and liver fibrosis are scarce. We aimed to explore endocan levels in relation to different stages of liver diseases, such as NAFLD, as determined with fatty liver index (FLI) and liver fibrosis, as assessed with BARD score.

**Methods:**

A total of 147 participants with FLI≥60 were compared with 64 participants with FLI <30. An FLI score was calculated using waist circumference, body mass index, gamma-glutamyl transferase and triglycerides. Patients with FLI≥60 were further divided into those with no/mild fibrosis (BARD score 0-1 point; n=23) and advanced fibrosis (BARD score 2-4 points; n=124). BARD score was calculated as follows: diabetes mellitus (1 point) + body mass index≥28 kg/m2 (1 point) + aspartate amino transferase/alanine aminotransferase ratio≥0.8 (2 points).

**Results:**

Endocan was independent predictor for FLI and BARD score, both in univariate [OR=1.255 (95% CI= 1.104-1.426), P=0.001; OR=1.208 (95% CI=1.029-1.419), P=0.021, respectively] and multivariate binary logistic regression analysis [OR=1.287 (95% CI=1.055-1.570), P=0.013; OR=1.226 (95% CI=1.022-1.470), P=0.028, respectively]. Endocan as a single predictor showed poor discriminatory capability for steatosis/fibrosis [AUC=0.648; (95% CI=0.568-0.727), P=0.002; AUC= 0.667 (95% CI=0.555-0.778), P=0.013, respectively], whereas in a Model, endocan showed an excellent clinical accuracy [AUC=0.930; (95% CI=0.886-0.975), P<0.001, AUC=0.840 (95% CI=0.763-0.918), P<0.001, respectively].

**Conclusions:**

Endocan independently correlated with both FLI and BARD score. However, when tested in models (with other biomarkers), endocan showed better discriminatory ability for liver steatosis/fibrosis, instead of its usage as a single biomarker.

## Abstract (sr)

**Uvod:**

Nema mnogo studija koje su ispitivale vrednosti endokana kod obolelih od nealkoholne steatoze i fibroze jetre. Na cilj je bio da se ispita nivo endokana u različitim stadijumima oboljenja jetre, kao to su nealkoholna steatoza jetre, predstavljena indeksom masne jetre (FLI) i fibroza jetre, predstavljena BARD skorom.

**Metode:**

Ukupno 147 učesnika sa FLI≥60 poređeno je sa 64 učesnika sa FLI <30. FLI skor je izračunat koristeći vrednosti obim struka, indeksa telesne mase, aktivnosti gama-glutamil transferaze i vrednosti triglicerida. Ispitanici sa FLI≥60 su dalje podeljeni u 2 grupe: bez fibroze/blaga fibroza (BARD skor 0-1 poen; n=23) i uznapredovala fibroza (BARD skor 2-4 poena; n=124). BARD skor je računat na sledeći način: šećerna bolest (1 poen) + indeks telesne mase≥28 kg/m2 (1 poen) + odnos aspartat aminotransferaza/alanin aminotransferaza≥0,8 (2 poena).

**Rezultati:**

Endokan je nezavisan prediktor FLI i BARD skora, kako u univarijantnoj [OR=1,255 (95% CI=1,104-1,426), P=0,001; odnosno OR=1,208 (95% CI=1,029-1,419), P=0,021], tako i u multivarijantnoj binarnoj logističkoj regresionoj analizi [OR=1.287 (95% CI=1,055-1,570), P=0,013; odnosno OR=1,226 (95% CI=1,022-1,470), P=0,028]. Endokan kao samostalan prediktor pokazao je slabu diskriminatornu moć za steatozu/fibrozu jetre [AUC=0,648; (95% CI=0,568-0,727), P=0,002; odnosno AUC=0,667 (95% CI=0,555-0,778), P=0,013], ali je u Modelu pokazao odličnu kliničku tačnost [AUC=0,930; (95% CI=0,886-0,975), P<0,001; odnosno AUC=0,840 (95% CI=0,763-0,918), P<0,001].

**Zaključak:**

Endokan je nezavisno povezan kako sa FLI, tako i sa BARD skorom. Ipak, u modelu (sa drugim biomarkerima), endokan je pokazao bolju diskriminatornu sposobnost za steatozu/fibrozu jetre.

## Introduction

Endocan is a proteoglycan with increased expression in endothelial cells during even the first stage of atheroslerosis [1], which makes this biomarker a convenient parameter of atherosclerosis risk. Not only that it is secreted by endothelial cells, but endocan also stimulates these cells to secrete other inflammation markers (i.e. cytokines), contributes to leukocytes migration, and has an impact on permeability of blood vessels [2] [3] [4], all of which further aggravate inflammation and increase cardiovascular disease (CVD) risk burden.

In addition to CVD [1], higher levels of this inflammation biomarker are also reported in states with diminished insulin sensitivity, such as type 2 diabetes [5] [6], polycystic ovary syndrome [7], nonalcoholic fatty liver disease (NAFLD) [8], which raises the question whether endocan might represent the link between inflammation in all these mentioned disorders and CVD.

The NAFLD is the most frequent manifestation of hepatic disorders [9]. It represents the increased accumulation of lipids which can trigger inflammation [10], and lesions of hepatocytes, that can consequently progress into fibrosis [11]. Even more, if not treated fibrosis can further progress into cirrhosis and hepatocellular carcinoma [11].

Increased inflammation and oxidative stress are observed in NAFLD [12] [13] [14]. These patho physiological mechanisms can stimulate the synthesis of collagen and induce hepotocytes apoptosis [15]. Moreover, the impairment of lipids and lipoprotein concentration was also shown in liver steatosis and fibrosis [14] [16], paralell with the increased prevalence of obesity and type 2 diabetes mellitus [17] [18] [19] [20].

On the other hand, endocan is not investigated thoroughly in different stages of liver diseases, such as NAFLD and liver fibrosis. Even more, a few studies that examined the concentration of this biomarker in fatty liver disease are conflicting [8] [21] [22] [23]. Since NAFLD represents an independent risk factor for CVD [24], we aimed to explore serum endocan concentration in relation to different stages of liver diseases, such as NAFLD, as determined with fatty liver index (FLI) and liver fibrosis, as assessed with BARD score.

## Materials and Methods

### Subjects

This case-control study included a total of 147 participants with fatty liver diagnosed with FLI≥60, who were compared with 64 controls, without fatty liver (i.e., FLI <30). The participants were consecutively recruited when visiting the Primary Health Care Center in Podgorica, Montenegro, for evaluation of metabolic parameters. The survey was conducted during a period from May to July 2017.

The inclusion criteria for participants were NAFLD diagnosed with FLI≥60 [25] with or without type 2 diabetes. Diabetes was diagnosed by American Diabetes Association Standards of Diabetes Care [26].

Algorithm FLI is calculated by waist circumference (WC), body mass index (BMI), gamma-glutamyl transferase (GGT) and triglycerides (TG) using equation [25]:

FLI = (e^0.953^ ^× loge (TG) + 0.139 × BMI + 0.718 × loge (GGT)^ ^+ 0.053 × WC − 15.745^) /(1+e^0.953 × loge (TG) + 0 .139 × BMI^ ^+ 0.718 × loge (GGT) + 0.053 × WC − 15.745^) × 100.

An FLI score is regarded to be a reliable algorithm for NAFLD assessment in general population, showing good specificity and sensitivity for NAFLD when diagnosed by abdominal ultrasonography, whereas FLI≥60 rules in, and FLI <30 rules out this metabolic disorder [25] [27] [28].

Thereafter, only those participants with NAFLD with FLI≥60 were further analyzed for potential liver fibrosis. Namely, BARD score [29] was calculated as follows: diabetes mellitus (1 point) + BMI≥28 kg/m^2^ (1 point) + aspartate aminotransferase (AST)/ alanine aminotransferase (ALT) ratio≥0.8 (2 points). Harrison et al. [29] showed that a BARD score shows a negative predictive value of 96% for liver fibrosis.

Accordingly, a total of 147 participants with FLI≥60 were stratified into two groups: those with no/mild fibrosis (i.e., BARD score 0-1 point; n=23) and the others with advanced fibrosis (i.e., BARD score 2-4 points; n=124).

Participants with 30≤ FLI <60, with alcohol consumption (>20 g/day), malignant diseases, type 1 diabetes, with a history of acute myocardial infarction and/or stroke in the last 6 months, renal disease other than diabetic nephropathy, thyroid dysfunction, high sensitivity C-reactive protein (hsCRP) >10 mg/L, and pregnant women were excluded from the examination.

Each participant signed an informed consent and the study protocol was approved by the Ethics Committee of the Primary Health Care Center in Podgorica. The study was performed in conformance with the Declaration of Helsinki ethical guidelines.

Anthropometric parameters (i.e., WC and BMI) were obtained by the same healthcare professional.

The blood pressure was measured after the patient's rest for 5 minutes. The average of the 3 measurements with sphygmomanometer and taken on the right arm was used.

### Methods

The phlebotomy was done between 7: 00 and 10:00 a.m. after an over night fast of at least 8 hours. Blood samples were taken in tubes with serum separator and clot activator, and each sample was left to clot within half an hour. Afterwards, the samples were centrifuged at 3000xg, at room temperature for 10 minutes. Sera were divided into aliquots and stored at -80 °C for determination of endocan and hsCRP, whereas one aliquot of each serum was analysed immediately after centrifugation for lipid parameters [i.e., total cholesterol (TC), TG, low density lipoprotein cholesterol (LDL-c), and high density lipoprotein cholesterol (HDL-c)], glucose, AST, ALT, GGT and creatinine. All these parameters were determined on Roche Cobas c501 chemistry analyzer (Roche Diagnostics GmbH, Mannheim, Germany). Serum endocan level was determined by using an enzyme-linked immunosorbent commercial assay (ab213776 -Human ESM1 ELISA Kit, Abcam, Cambridge, UK), whereas serum hsCRP levels were determined nephelometrically (Behring Nephelometer Analyzer, Marburg, Germany).

### Statistical analysis

Statistical analysis was done using SPSS 21.0 (SPSS Inc., Chicago, USA). Data distribution was tested by Shapiro Wilk test. Continuous variables were presented as median (interquartile range) and tested by Mann-Whitney test. Categorical variables were presented as absolute and relative frequencies and analyzed using the Chi-square test for contingency tables. Spearman's correlation analysis (r) was applied to examine the associations between FLI and BARD score with the clinical data. Furthermore, binary logistic regression analysis was applied in order to test the independent associations of endocan with FLI and BARD score. When performing logistic regression analysis, FLI <30 was used as a reference and FLI≥60 as risk categories, while BARD scores 0 and 1 point were used as reference (i.e., no/mild fibrosis) and BARD scores 2, 3 and 4 points were used as risk categories (i.e., advanced fibrosis). Multivariate logistic regression analysis was used to investigate possible independent predictions of endocan on the presence of fatty liver, as well as on advanced fibrosis. Data from binary logistic regression analysis were showed as odds ratio (OR) and 95% confidence interval (CI). Receiver operating characteristic (ROC) analysis and the area under the ROC curve (AUC) were used to test the predictive ability of endocan, solely and in a model, for identifying patients with fatty liver and advanced fibrosis. Clinical accuracy of endocan was analysed according to Swets [30]. The P value less than 0.05 was considered as statistically significant.

## Results

Clinical data of participants are summarized in Table 1. Significantly more men were found in the group with fatty liver than in the group without it. Individuals with fatty liver displayed greater BMI, WC, as well as higher prevalence of type 2 diabetes, as compared with individuals without fatty liver. More subjects with fatty liver used antihyperglycemic, insulin and antihypertensive therapy, than those without fatty liver. Also, they had significantly higher glucose, HbA1c, TG, ALT, GGT, hsCRP and endocan levels, but lower HDL-c levels than individuals without fatty liver.

**Table 1:** Clinical data and endocan levels in patients without and with fatty liver Data are given as median (interquartile range) and compared by Mann-Whitney test. BMI – Body mass index; WC – Waist circumference; SBP – Systolic blood pressure; DBP – Diastolic blood pressure; FLI – Fatty liver index; HbA1c – Glycated hemoglobin; TC – Total cholesterol; HDL-cholesterol – High density lipoprotein cholesterol; LDLcholesterol – Low density lipoprotein cholesterol; TG – Triglycerides; AST – Aspartate aminotransferase; ALT – Alanine aminotransferase; GGT – Gamma-glutamyl transferase; HsCRP – High-sensitivity C-reactive protein

	Without fatty liver (FLI <30)	Fatty liver (FLI≥60)	P
N (male, %)	64 (14%)	147 (47%)	<0.001
Age, years	60 (52–65)	62 (57–68)	0.051
BMI, kg/m^2^	24.1 (23.1–25.3)	31.6 (29.9–33.8)	<0.001
WC, cm	85 (81–89)	105 (100–111)	<0.001
SBP, mmHg	136 (126–151)	130 (125–145)	0.243
DBP, mmHg	86 (77–94)	83 (76–92)	0.345
Diabetes, n (%)	10 (16%)	75 (51%)	<0.001
Smokers, n (%)	15 (23%)	23 (16%)	0.176
Antihyperglycemics, n (%)	7 (11%)	65 (44%)	<0.001
Insulin therapy, n (%)	2 (3%)	22 (15%)	0.016
Antihypertensives, n (%)	31 (48%)	111 (76%)	<0.001
Hypolipidemics, n (%)	18 (28%)	58 (39%)	0.122
FLI	16 (13–22)	83 (70–91)	<0.001
Glucose, mmol/L	5.5 (5.3–5.8)	6.4 (5.5–8.3)	<0.001
HbA1c, %	5.4 (5.2–5.7)	6.0 (5.5–7.2)	<0.001
HbA1c, mmol/mol	36 (33–39)	42 (37–55)	<0.001
TC, mmol/L	5.75 (4.75–6.54)	5.83 (4.87–6.93)	0.475
HDL-cholesterol, mmol/L	1.73 (1.53–2.03)	1.19 (1.00–1.40)	<0.001
LDL-cholesterol, mmol/L	3.10 (2.52–4.025)	3.45 (2.62–4.53)	0.254
TG, mmol/L	1.17 (0.94–1.43)	2.25 (1.71–2.95)	<0.001
AST, U/L	20 (17–23)	20 (17–24)	0.646
ALT, U/L	17 (11–22)	22 (17–32)	<0.001
GGT, U/L	12 (9–16)	22 (16–31)	<0.001
HsCRP, mg/L	0.52 (0.32–0.98)	1.70 (0.77–3.11)	<0.001
Endocan, ng/L	27.8 (17.6–40.9)	38.8 (21.6–89.5)	0.002

The correlation coefficients from Spearman's correlation analysis between FLI and clinical data were showed in Table 2. BMI, WC, glucose, HbA1c, TG, ALT, GGT and hsCRP showed positive, but HDLc negative correlations with FLI.

**Table 2:** Spearman’s correlation analysis of clinical markers and FLI Data age given as coefficients of correlation Rho (ρ). BMI-Body mass index; WC-Waist circumference; SBP-Systolic blood pressure; DBP-Diastolic blood pressure; HbA1c-Glycated hemoglobin; TC-Total cholesterol; HDL-cholesterol-High density lipoprotein cholesterol; LDL-cholesterol-Low density lipoprotein cholesterol; TG-Triglycerides; AST-Aspartate aminotransferase; ALT-Alanine aminotransferase; GGT Gammaglutamyl transferase; HsCRP-High-sensitivity C-reactive protein

Variable	ρ	P
Age, years	0.121	0.079
BMI, kg/m^2^	0.840	<0.001
WC, cm	0.902	<0.001
SBP, mmHg	-0.038	0.578
DBP, mmHg	-0.052	0.456
Glucose, mmol/L	0.435	<0.001
HbA1c, %	0.397	<0.001
TC, mmol/L	-0.026	0.709
HDL-cholesterol, mmol/L	-0.504	<0.001
LDL-cholesterol, mmol/L	-0.028	0.685
TG, mmol/L	0.582	<0.001
AST, U/L	0.109	0.115
ALT, U/L	0.390	<0.001
GGT, U/L	0.639	<0.001
HsCRP, mg/L	0.413	<0.001
Endocan, ng/L	0.203	0.005

Binary logistic regression analysis was applied to determine in-depth associations of endocan and the presence of fatty liver disease. In univariate analysis, endocan showed significant relation with fatty liver disease [OR=1.255 (95% CI=1.104-1.426), P=0.001]. Negelkerke R^2^ in univariate regression, analysis was 0.125. Markers significantly correlated with FLI in non-parametric Spearman’s correlation analysis (i.e., HbA1c, HDL-c, ALT, hsCRP and endocan), but not used for FLI calculation, as well as demographic characteristics significantly different between FLI groups (i.e., gender, antihyperglycemic, insulin and antihypertensive therapies) were used in Model to test the independent prediction of endocan for fatty liver disease. Endocan was shown to be an independent predictor for fatty liver [OR=1.287 (95% CI=1.055-1.570), P=0.013]. Nagelkerke R^2^ of 0.656 demonstrated that even 65.6% of variation in fatty liver disease occurrence could be explained by this Model.

**Figure 1:**
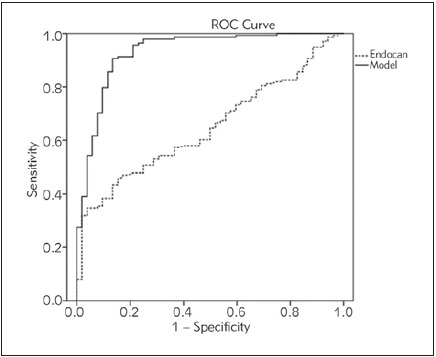
ROC curves of endocan and selected Model discriminatory capabilities towards presence of fatty liver

ROC analysis was used to discriminate patients with fatty liver from those without fatty liver (Figure 1). Endocan as a single predictor showed poor discriminatory capability [AUC=0.648; (95% CI=0.568-0.727), P=0.002]. Specificity of this test was 96.16%, but sensitivity was 34.78%. On the contrary, when tested in the Model, endocan showed an excellent clinical accuracy [AUC=0.930; (95% CI=0.886-0.975)] with sensitivity of 90.58% and specificity of 86.54%.

Only patients with fatty liver (FLI≥60) were further tested for possible presence of advanced fibrosis. Their characteristics were given in Table 3. Significantly more women were found among those with advanced than with no/mild fibrosis. Patients with advanced fibrosis were older, had higher BMI, and used frequently more antihyperglycemic and antihypertensive therapies than those with no/mild fibrosis. Also, significantly more of them had diabetes. Glucose, HbA1c, ALT and endocan levels were higher in those with advanced fibrosis than in individuals with no/mild fibrosis.

**Table 3:** Clinical data and endocan levels in patients without and with advanced fibrosis Data are given as median (interquartile range) and compared by Mann-Whitney test

	No/mild fibrosis	Advanced fibrosis	P
N (male, %)	23 (70%)	124 (43%)	0.018
Age, years	56 (51–61)	64 (58–70)	<0.001
BMI, kg/m^2^	29.7 (28.1–33.3)	31.8 (30.3–34.3)	0.020
WC, cm	105 (99–107)	107 (101–112)	0.233
SBP, mmHg	139 (126–146)	130 (125–144)	0.586
DBP, mmHg	84 (80–92)	83 (76–90)	0.314
Diabetes, n (%)	4 (17%)	71 (57%)	<0.001
Smokers, n (%)	5 (22%)	18 (15%)	0.381
Antihyperglycemics, n (%)	3 (13%)	62 (50%)	0.001
Insulin, n (%)	3 (13%)	19 (15%)	0.778
Antihypertensives, n (%)	12 (52%)	99 (80%)	0.005
Hypolipidemics, n (%)	8 (36%)	50 (40%)	0.618
Glucose, mmol/L	5.4 (5.2–6.1)	6.7 (5.8–8.5)	<0.001
HbA1c, %	5.5 (5.3–5.9)	6.3 (5.6–7.3)	0.002
HbA1c, mmol/mol	37 (34–41)	45 (37–56)	0.002
TC, mmol/L	6.59 (5.04–7.79)	5.78 (4.87–5.79)	0.209
HDL-cholesterol, mmol/L	1.21 (1.01–1.32)	1.19 (0.99–1.34)	0.841
LDL-cholesterol, mmol/L	3.94 (3.07–4.86)	3.40 (2.60–4.37)	0.151
TG, mmol/L	2.36 (1.73–2.84)	2.20 (1.70–2.97)	0.821
AST, U/L	20 (19–24)	20 (17–25)	0.443
ALT, U/L	31 (25–39)	21 (16–28)	<0.001
GGT, U/L	23 (16–35)	22 (15–30)	0.552
HsCRP, mg/L	1.81 (0.46–2.94)	1.67 (0.80–3.11)	0.362
Endocan, ng/L	26.0 (14.2–44.3)	44.2 (22.8–92.7)	0.013

Years of age, glucose, HbA1c, and endocan correlated significantly positively with BARD score (Table 4). On the contrary, ALT and TG correlated significantly negatively with BARD score.

**Table 4:** Spearman’s correlation analysis of clinical markers and BARD Data age given as coefficients of correlation Rho (ρ). BMI – Body mass index; WC – Waist circumference; SBP – Systolic blood pressure; DBP Diastolic blood pressure; HbA1c – Glycated hemoglobin; TC – Total cholesterol; HDL-cholesterol – High density lipoprotein cholesterol; LDL-cholesterol – Low density lipoprotein cholesterol; TG – Triglycerides; AST – Aspartate aminotransferase; ALT – Alanine aminotransferase; GGT – Gamma-glutamyl transferase; HsCRP – High-sensitivity C-reactive protein

Variable	ρ	P
Age, years	0.419	<0.001
BMI, kg/m^2^	0.145	0.080
WC, cm	0.052	0.529
SBP, mmHg	0.023	0.782
DBP, mmHg	0.017	0.842
Glucose, mmol/L	0.349	<0.001
HbA1c, %	0.307	<0.001
TC, mmol/L	-0.142	0.085
HDL-cholesterol, mmol/L	0.148	0.074
LDL-cholesterol, mmol/L	-0.142	0.086
TG, mmol/L	-0.195	0.018
AST, U/L	-0.049	0.522
ALT, U/L	-0.461	<0.001
GGT, U/L	-0.131	0.110
HsCRP, mg/L	0.062	0.457
Endocan, ng/L	0.217	0.010

Binary logistic regression analysis was used to test the associations of endocan with the presence of advanced fibrosis. In univariate analysis odds ratio for endocan was OR=1.208 (95% CI=1.029-1.419), P=0.021, R^2^ =0.088. In multivariate analysis, in the Model that was consisted of gender, ages, TG, antihypertensive therapy and endocan, the latest was shown to be the independent predictor of advanced fibrosis [OR=1.226 (95% CI=1.022-1.470), P=0.028]. Clinical markers that entered the Model were continuous variables which showed significant correlation with BARD score in Spearman's correlation analysis (Table 4) and categorical data significantly different between no/mild vs. advanced fibrosis (Table 3). Nagelkerke R^2^ for the Model was 0.330 which shows that 33% of variation in the presence of advanced fibrosis could be explained by this Model.

**Figure 2:**
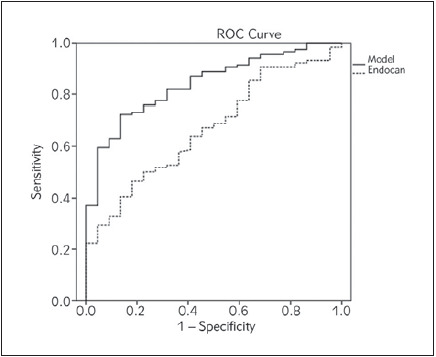
ROC curves of endocan and selected Model discriminatory capabilities towards presence of advanced fibrosis

ROC analysis showed that curve for the Model (Figure 2) had an excellent discriminatory capability [AUC=0.840 (95% CI=0.763-0.918), P<0.001] towards advanced fibrosis with specificity of 86.36% and sensitivity of 72.41%. On the other hand, endocan itself has poor discriminatory ability towards advanced fibrosis [AUC=0.667 (95% CI=0.555-0.778), P=0.013] with specificity 81.82% and sensitivity 46.55%.

## Discussion

Our study shows that endocan levels were higher in NAFLD (as assessed with FLI), as well as in advanced fibrosis (as assessed with BARD score), as compared with controls. Importantly, endocan independently correlated with both, FLI and BARD score.

Only a small number of previous studies examined this biomarker in NAFLD, and showed contradictory results [8] [21] [22] [23]. Tok et al. [22] showed lower endocan concentration in 38 patients with NAFLD, whereas Dallilo et al. [8] reported vice versa (i.e., its higher levels in 19 patients with NAFLD and 32 with type 2 diabetes mellitus with NAFLD), as compared with controls. On the contrary, Ustyol et al. [23] found no difference in serum endocan levels between obese participants with and without NAFLD, as compared with controls in pediatric population.

Also, previous study [31] investigated serum endocan in patients with liver cirrhosis and found only higher level in patients with decompensated cirrhosis, but no difference between compensated cirhhosis and healthy controls was observed.

Additionally, another study showed that patients with NAFLD and coronary artery disease displayed higher serum endocan levels as compared with patients with NAFLD, but without coronary artery disease [21].

Possible discrepancies in those results might be explained by the fact that sample size of participants differed between the studies. In our study we included a relatively larger sample size than previous studies did (i.e., a total of 147 participants with NAFLD), and only adult Caucasians (i.e., Montenegrin) population [8] [22] [23].

Endocan is highly expressed in endothelial cell injury [1] with its up-regulation observed in the presence of proangiogenic molecules and proinflammatory cytokines [2]. The pathological accumulation of lipids in NAFLD triggers inflam mation [10], which can lead to hepatocytes dysfunction. Additionally, paralell with the liver fibrosis and with the processes of its repairment, increased production of extracellular matrix proteins occurs [11]. Since proteoglycans are constitute of the extracellular matrix, acting as its structural components [32], this might explain the higher level of endocan, as one of the proteoglycans, in liver fibrosis.

Considering the fact that NAFLD is an independent predictor for CVD [24], not only that endocan might reflect the severity of liver failure, but further prospective studies are necessary to explore the causal link between high endocan level, liver steatosis/fibrosis and CVD.

In our study median endocan level in patients with NAFLD was 38.8 ng/L (21.6-89.5), whereas in advanced fibrosis was 44.2 ng/L (22.8-92.7), as compared with non-NAFLD group [median 27.8 ng/L (17.6-40.9)], thus presuming its increase with progression of liver disease.

Although an independent association between liver steatosis/fibrosis and serum endocan level is shown in the current study, this proteoglycan seems to be more convenient in the diagnostic evaluation of these liver disorders, in combination with other markers, instead of its usage alone, as a single biomarker. Namely, in our study endocan showed poor discriminatory capability (AUC=0.648) for NAFLD as a single predictor. On the contrary, when tested in the Model [i.e., variables that entered the Model were: gender, HbA1c, insulin, antihyperglycemic and antihypertensive therapies (categorical variables), and HDL-c, ALT, hsCRP, endocan (continuous variables)], endocan showed an excellent clinical accuracy (AUC=0.930) with sensitivity of 90.58% and specificity of 86.54%.

We obtained the similar results when evaluating the diagnostic accuracy of endocan in liver fibrosis. Namely, endocan itself showed poor discriminatory ability for advanced fibrosis (AUC=0.667). However, when tested in the Model [i.e., variables that entered the Model: gender, antihypertensive therapy (categorical variables) and ages, TG, endocan (continuous variables)], endocan showed an excellent discriminatory capability (AUC=0.840) for advanced fibrosis, with specificity of 86.36% and sensitivity of 72.41%.

Our previous studies have also demonstrated the benefits of multimarker approach in better discrimination of individuals with liver steatosis [12] [13] [14]. Namely, an independent relationship between FLI and insulin resistance (i.e. HOMA-IR) and inflammation (i.e. hsCRP) was recorded in the cohort of postmenopausal women [13]. Also, ALT was shown to be independently correlated with FLI in both genders [12] in a large Montenegrin population sample, whereas HDL-c and malondialdehyde independently correlated with FLI in the cohort of patients with type 2 diabetes mellitus [14]. However, when tested in model with other lipid, inflammation and oxidative stress biomarkers, the discriminative ability for liver steatosis development was significantly enhanced [14]. These mentioned results point out that in addition to traditional risk factors, multimarker approach including cluster of different biomarkers may significantly improve the timely identification of those patients with high risk of liver steatosis. Moreover, in another study we have also reported that older age and higher HDL-c are independently correlated with advanced liver fibrosis assessed with the BARD score, suggesting that further examination of enzymes involved in lipoprotein metabolism could be perspective for revealing the causal association between lipid parameters and liver fibrosis [16].

The main disadvantage of this study is its crosssectional design which limits us to conclude the cause-effect between high endocan and liver steatosis/fibrosis. Additionally, we were not able to use imaging diagnostic procedures, but simple and easy obtained algorithms, like previous studies did [28] [33]. However, the 2016 European Association for the Study of the Liver (EASL), European Association for the Study of Diabetes (EASD), and European Association for the Study of Obesity (EASO), recommended the usage of the FLI as one of the best validated steatosis scores for screening studies in large samples [28]. Additionally, BARD score is shown to be useful for ruling out advanced fibrosis, thus reducing the need for liver biopsies in NAFLD patients [34] [35].

## Conclusion

Higher serum endocan levels were observed in NAFLD (as determined with FLI) and in advanced fibrosis (as determined with BARD score), as compared with controls. Importantly, endocan was independently correlated with both, FLI and BARD score. However, when tested in models (with other biomarkers), this proteoglycan showed better discriminatory ability for liver steatosis/fibrosis, instead of its usage alone, as a single biomarker.

*Acknowledgement*.This work was financially supported in part by a grant from the Ministry ofScience, Montenegro and the Ministry of Education, Science and Technological Development, Republic of Serbia (project number 175035).

## Conflict of interest statement

The authors state that they have no conflicts of interest regarding the publication of this article.
